# Effect of Exercise on Inflamed Psoas Muscle in Women with Obesity: A Pilot Prospective ^18^F-FDG PET/CT Study

**DOI:** 10.3390/diagnostics11020164

**Published:** 2021-01-24

**Authors:** Kisoo Pahk, Eung Ju Kim, Chanmin Joung, Hyun Woo Kwon, Hong Seog Seo, Sungeun Kim

**Affiliations:** 1 Department of Nuclear Medicine, Korea University Anam Hospital, Seoul 02841, Korea; kisu99@korea.ac.kr (K.P.); hnwoo@korea.ac.kr (H.W.K.); 2 Department of Cardiovascular Center, Korea University Guro Hospital, Seoul 08308, Korea; withnoel@empal.com; 3 Institute for Inflammation Control, Korea University, Seoul 02841, Korea; joungchanmin@korea.ac.kr

**Keywords:** obesity, skeletal muscle, psoas muscle, inflammation, exercise, positron-emission tomography

## Abstract

Obesity increases inflammation in skeletal muscle thereby promoting systemic inflammation which leads to increased risk of cardiometabolic disease. This prospective study aimed to evaluate whether the metabolic activity of psoas muscle (PM) was associated with systemic inflammation, and whether physical exercise could reduce the PM metabolic activity evaluated by ^18^F-fluorodeoxyglucose (FDG) positron emission tomography/computed tomography (PET/CT) in women with obesity. A total of 23 women with obesity who participated in a 3-month physical exercise program were enrolled. ^18^F-FDG PET/CT was performed before the start of the program (baseline) and after completion of the program. The maximum standardized uptake value of psoas muscle (PM SUVmax) was used for the PM metabolic activity. The SUVmax of spleen and bone marrow, and the high-sensitivity C-reactive protein were used to evaluate the systemic inflammation. At baseline, PM SUVmax was strongly correlated with the systemic inflammation. The exercise program significantly reduced the PM SUVmax, in addition to adiposity and systemic inflammation. Furthermore, we found that the association between PM SUVmax and the systemic inflammation disappeared after completion of the exercise program. In women with obesity, PM SUVmax, assessed by ^18^F-FDG PET/CT, was associated with obesity-induced systemic inflammation and exercise reduced the PM SUVmax and eliminated its association with systemic inflammation.

## 1. Introduction

Obesity is becoming a global epidemic health concern with a continuously increasing prevalence [1]. Furthermore, it is an independent risk factor for cardiometabolic disease, such as cardiovascular disease (CVD), diabetes, and hypertension, which is the leading cause of death globally [1,2,3].

In the progress of obesity, increased visceral obesity seems to shift skeletal muscle metabolism towards an inflammatory status [4,5]. Inflamed skeletal muscle secrete large numbers of pro-inflammatory cytokines, thereby accelerating inflammatory cell infiltration into skeletal muscle, which can further promote insulin resistance and increase the risk of atherosclerotic plaque rupture, which collectively result in a higher risk of cardiometabolic disease [6,7,8]. In addition, skeletal muscle accounts for 80% of insulin-stimulated glucose uptake in the whole-body and has been regarded as the most important organ for glucose homeostasis [4,9,10]. Thus, inflamed skeletal muscle could be central to the development of cardiometabolic disease.

Glucose metabolism is upregulated in inflammatory cells, such as macrophages, which is a hallmark of the inflammatory process. ^18^F-fluorodeoxyglucose (FDG) positron emission tomography/computed tomography (PET/CT), a non-invasive glucose imaging technique, is well-known to evaluate the inflammatory activity of inflamed tissue, such as vulnerable atherosclerotic plaques [11,12]. As macrophages are also increased in inflamed skeletal muscle [6,7,8], it would also be feasible to use the ^18^F-FDG PET/CT to evaluate the inflammatory activity of inflamed skeletal muscle.

For the assessment of skeletal muscle metabolism, psoas muscle (PM) mass has been widely used as a representative index of skeletal muscle mass due to its relative independence from physical activity levels, compared to appendicular muscle mass [13]. Furthermore, recently, Kim et al. reported that ^18^F-FDG uptake of PM was increased in populations with metabolic syndrome, which is strongly associated with obesity [14]. Thus, we postulated that the metabolic activity of PM, assessed by ^18^F-FDG PET/CT, could reflect the inflammatory activity in obesity-induced skeletal muscle inflammation.

Chronic exercise is known to have a protective effect on cardiometabolic disease, partly through its anti-inflammatory effect on chronic systemic inflammation which can be synergistically induced by inflammatory activity in both visceral fat and skeletal muscle [8,15]. Several previous studies have reported that physical exercise could decrease visceral fat mass with an inhibition of inflammatory activity in visceral fat via the use of various imaging modalities such as CT, magnetic resonance imaging (MRI), or ^18^F-FDG PET/CT in populations with obesity [16,17,18]. In addition to visceral fat, physical exercise also has been known to show anti-inflammatory effects on skeletal muscle in both human and animal studies [19,20]. However, few non-invasive imaging studies have been conducted on the change of skeletal muscle inflammatory activity in response to regular exercise in populations with obesity.

The aim of this study was to investigate whether the metabolic activity of PM, evaluated by ^18^F-FDG PET/CT, was associated with systemic inflammation, and whether physical exercise could attenuate the metabolic activity of PM in women with obesity.

## 2. Materials and Methods

### 2.1. Study Participants

**Figure 1:**
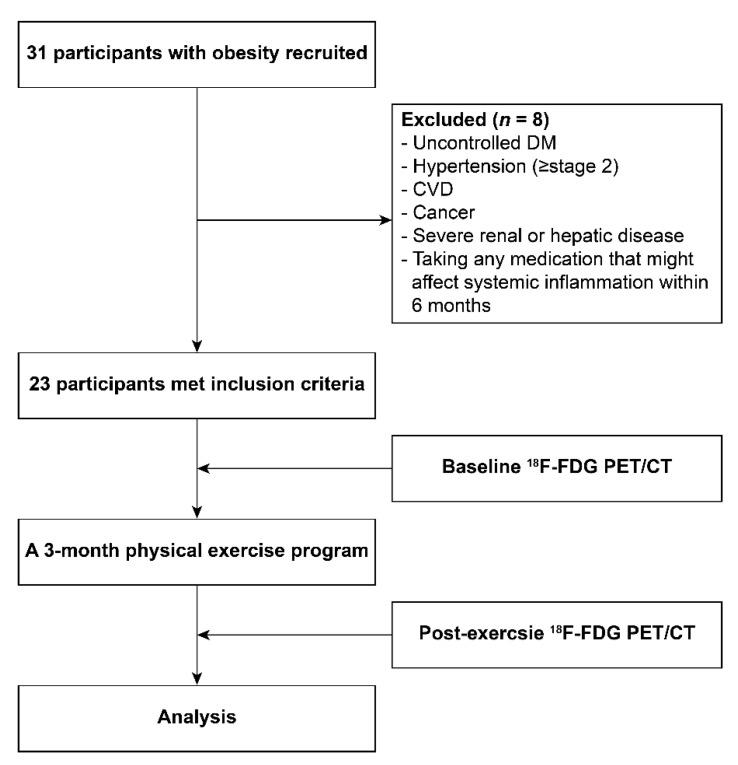
Flowchart of study design. DM, diabetes mellitus; CVD, cardiovascular disease; ^18^F-FDG PET/CT, ^18^F-fluorodeoxyglucose (FDG) positron emission tomography/computed tomography.

From June 2008 to March 2009, a total of 31 participants with obesity were enrolled from a community health center, prospectively. Obesity is determined as a body mass index (BMI) of over 25 kg/m^2^, according to the obesity guideline for a Korean population [21]. Participants with uncontrolled diabetes mellitus, hypertension (≥stage 2), cardiovascular disease, malignancy, severe renal or hepatic disease, and those taking any medication that might affect systemic inflammation within 6 months of this study were excluded. Finally, in total, 23 women with obesity were enrolled in this study (Figure 1) and all provided written informed consent. This study was approved by the institutional review board of Korea University Guro Hospital (approval No. GR0888-005).

### 2.2. Study Design

The exercise program was performed 5 times a week for 3 months under supervision of trainer and without diet restriction. All evaluation parameters such as anthropometric and clinical laboratory parameters were measured before the initiation of exercise program (baseline) and after 3 months of exercise program (post-exercise). Participants also underwent ^18^F-FDG PET/CT at baseline and post-exercise.

### 2.3. Exercise Program

The exercise program was composed of aerobic and muscle-resistant training, according to the recommendation of the American College of Sports Medicine (ACSM) and the American Heart Association (AHA) the program was reported to have a beneficial effect on health [22]. Aerobic exercise consisted of 30 min of moderate-intensity activity (walking at very brisk pace; 4 mph) followed by 20 min of vigorous-intensity (running at 7 mph). Intensity between 3 and 6 metabolic equivalents (METs) was defined as moderate-intensity and above 6 METs was defined as vigorous-intensity, respectively [22]. Subsequent muscle-resistant training consisted of 8 to 10 exercises involving the major muscles with 8 to 12 repetitions [22].

### 2.4. Anthropometric and Clinical Laboratory Parameters

BMI was calculated as weight/height squared (kg/m^2^). Waist and hip circumference was measured at midway between the lowest border of the rib cage and iliac crest in the sitting position and measured at the largest circumference over the hips in standing position, respectively. All blood samples were collected after overnight fasting and analyzed by using a chemistry analyzer (Hitachi 747, Hitachi, Tokyo, Japan). Blood pressure was measured by using an automated cuff (OMRON M10-IT, OMRON Healthcare, Kyoto, Japan).

### 2.5. ^18^F-FDG PET/CT Protocol

All participants undertook ^18^F-FDG PET/CT after overnight fasting to keep a blood glucose level under 180 mg/dL. The scan was started 60 min after the injection of 5.29 MBq/kg of ^18^F-FDG intravenously with a dedicated PET/CT scanner (GEMINI TF, Philips Medical Systems, Cleveland, OH, USA). The CT scan (120 kVp, 50 mA, 4 mm thickness) was used for attenuation correction from the skull to proximal thigh. The PET scan (18 cm axial field of view with 4.4 mm spatial resolution) was acquired immediately after the CT scan. All images were reconstructed with an iterative algorithm (three-dimensional row-action maximum likelihood algorithm).

### 2.6. Image Analysis

Images were analyzed by two expert blinded nuclear medicine physicians (K.P. and S.K.) on a dedicated workstation (Extended Brilliance Workspace version 3.5, Philips Healthcare, Eindhoven, Netherlands). For the evaluation of the metabolic activity of targeted region, regions of interest (ROIs) were located on the targeted region, and standardized uptake value (SUV) was calculated as follows:

SUV = Tracer activity (ROI) (MBq/mL)/Injected dose (MBq)/Total body weight (g)

To measure the metabolic activity of PM, the boundary of PM was identified by CT images at L4 spine level, as previously described [23]. Next, ROIs were manually placed on both right and left PM and their corresponding area and maximum SUV (SUVmax) were obtained. Representative PM area was defined as the average value of right and left PM area. Representative PM SUVmax, was defined as the average value of right and left PM SUVmax. Metabolic activities of spleen and bone-marrow (BM) assessed by ^18^F-FDG PET/CT could reflect the elevated myeloid activity arising from systemic inflammation, thereby being known for systemic inflammation surrogate markers [24,25]. To measure the metabolic activity of the spleen and BM, ROIs were located on the spleen and spinal BM at the level of L3 to L5 from all axial slices. Averaged SUVmax from those ROIs were used as representative spleen SUVmax and BM SUVmax, respectively [24].

### 2.7. Statistical Analysis

All data were expressed as the mean ± standard deviation. Normalcy was determined using the Shapiro–Wilk test. Differences between baseline and post-exercise were analyzed using Paired *t*-test or Wilcoxon signed-rank test. Student’s *t*-test or Mann–Whitney *U* test was employed to compare the two groups. Spearman’s correlation coefficient, univariate linear regression analysis, and partial correlation analysis were also used as statistical methods. The statistical power was set at 0.8. In multivariate analysis, Bonferroni correction was used. All data were analyzed using SPSS version 17.0 (SPSS Inc., Chicago, IL, USA) and MedCalc version 18.5 (MedCalc, Mariakerke, Belgium) with a significant *p*-value at <0.05.

## 3. Results

All participants successfully completed the planned physical exercise program and the baseline clinical characteristics of all participants are shown in Table 1. As shown in Table 2, physical exercise program exhibited profound reduction in body adiposity and blood pressure, as expected.

### 3.1. Association between PM SUVmax and Systemic Inflammation at Baseline

At baseline, PM SUVmax showed a significant positive correlation with hsCRP and spleen SUVmax, whereas it showed no significant correlation with PM area (Table 3). As shown in Table 4, univariate analysis showed that PM SUVmax was significantly associated with BMI, waist circumference, hip circumference, spleen SUVmax, and hsCRP. In further multivariate analysis, PM SUVmax was significantly associated with hsCRP. These results indicate that PM SUVmax is associated with systemic inflammation in women with obesity at baseline.

### 3.2. The Effect of Physical Exercise on PM SUVmax and Systemic Inflammation

**Figure 2:**
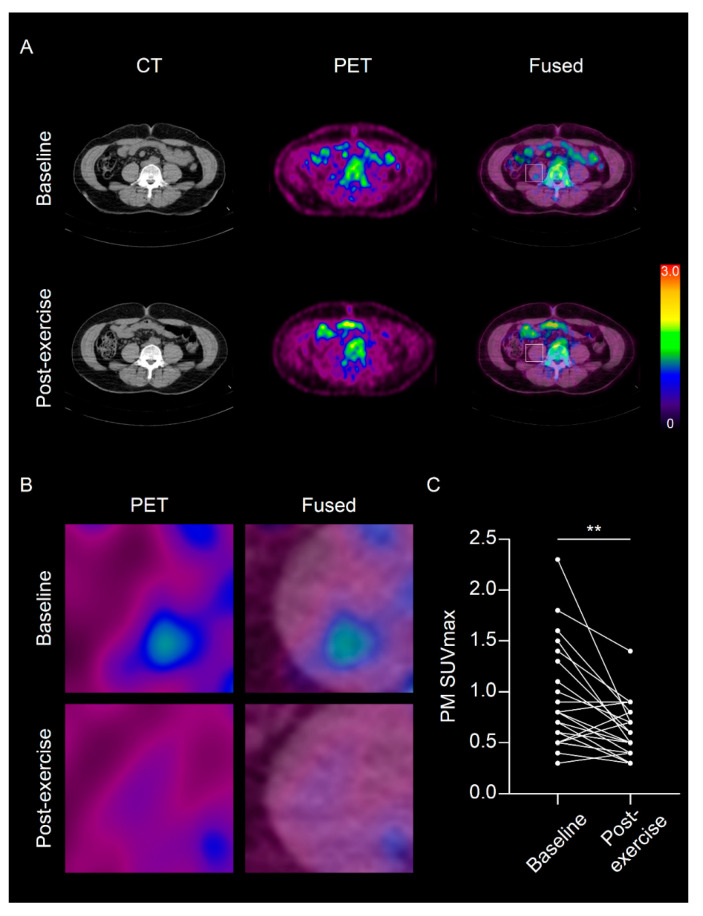
Physical exercise significantly attenuated the maximum standardized uptake value of psoas muscle (PM SUVmax). (**A**) Representative images of ^18^F-fluorodeoxyglucose (FDG) positron emission tomography/computed tomography (PET/CT) at baseline and post-exercise; (**B**) Magnified images of psoas muscle; (**C**) Change in PM SUVmax between baseline and post-exercise. ** *p* < 0.01.

A 3-month program of physical exercise significantly reduced PM SUVmax (0.93 ± 0.5 to 0.62 ± 0.27, *p* = 0.002, Figure 2A–C), whereas the PM area was not significantly changed (*p* = 0.402, Table 2). Furthermore, consistent with the change of PM SUVmax, systemic inflammation surrogate markers such as hsCRP, spleen SUVmax, and BM SUVmax were significantly reduced by physical exercise (Table 2).

### 3.3. Association between PM SUVmax and Systemic Inflammation after a 3-Month of Physical Exercise Program

After a 3-month physical exercise program, PM SUVmax was no longer correlated with both hsCRP and spleen SUVmax (Table 3). Furthermore, as shown in Table 5, the association between PM SUVmax and systemic inflammation surrogate markers had also disappeared. Thus, PM SUVmax was attenuated and was not related to systemic inflammation in women with obesity after they partook in physical exercise for 3 months.

## 4. Discussion

To the best of our knowledge, this is the first prospective study to explore the anti-inflammatory effect of physical exercise on the inflammatory metabolic activity of skeletal muscle using ^18^F-FDG PET/CT. In this study, we found that PM SUVmax, assessed by ^18^F-FDG PET/CT, showed a significant association with women with obesity and a 3-month program of physical exercise significantly decreased PM SUVmax and eliminated its association with systemic inflammation.

In obesity, inflamed skeletal muscle is composed of multiple heterogeneous cells such as myocytes, adipocytes, and inflammatory cells [8]. Accumulating evidence has reported that infiltrating inflammatory cells, such as macrophages, are the predominant inflammatory cells in inflamed skeletal muscle [26,27,28] with upregulated glucose uptake [29,30]. Regarding the glucose uptake of inflamed skeletal muscle, infiltrating macrophages mainly use insulin-independent glucose transporter-1 (GLUT-1) [30,31], whereas myocytes and adipocytes use insulin-dependent GLUT-4 for glucose uptake [6,8,10]. Thus, in inflamed skeletal muscle, since obesity-induced skeletal muscle inflammation is highly correlated with insulin resistance, macrophages show increased glucose uptake [29,30,31], whereas myocytes and adipocytes exhibit reduced glucose uptake [6,8,10]. Furthermore, infiltrating macrophages could also block the GLUT-4 expression in both myocytes and adipocytes in inflamed skeletal muscle, which collectively contribute to reduced glucose uptake in those cells [29,32,33]. Thus, we speculated that PM SUVmax could reflect the inflammatory metabolic activity of macrophages in obesity-driven inflamed skeletal muscle.

Chronic exercise has been known to decrease the number of circulating inflammatory cells thereby reducing the number of infiltrating macrophages in skeletal muscle [20,34]. Supporting the previous studies, this study showed that chronic exercise reduced both PM SUVmax and systemic inflammatory surrogate markers, such as hsCRP, spleen SUVmax, and BM SUVmax. Considering the causal relationship between systemic inflammation and inflamed skeletal muscle, the reduction in PM SUVmax could be accounted for by the reduced inflammatory activity of PM, which could be ascribed to the anti-inflammatory effect of chronic exercise.

However, although we found that chronic exercise could attenuate both PM SUVmax and systemic inflammation in this study, it is interesting that the presumed association between PM SUVmax and systemic inflammation in women with obesity disappeared after completion of a 3-month physical exercise program. Unfortunately, the detailed underlying mechanism remains unknown. However, in the present study, we found that PM SUVmax was reduced with physical exercise down to about 30% of the baseline. This could indicate the intrinsic level of PM SUVmax in metabolically healthy obesity, which might be explained by the weaker relationship with systemic inflammation. Further study is warranted to elucidate the underlying mechanism.

Recently, targeting obesity-induced skeletal muscle inflammation has been considered as a promising therapeutic strategy for obesity-induced cardiometabolic disease [8]. Furthermore, several pharmacological anti-inflammatory therapies remarkably alleviate obesity-induced skeletal muscle inflammation [35,36]. Considering this, evaluation of skeletal muscle inflammation for treatment response monitoring and risk stratification is crucial for management of patients with obesity. In this perspective view, muscle biopsy could be a gold standard to evaluate skeletal muscle inflammation. However, biopsy is an invasive procedure and sometimes difficult to perform in actual clinical practice. Thus, it is feasible to use PM SUVmax assessed by ^18^F-FDG PET/CT as a surrogate marker for reflecting obesity-induced skeletal muscle inflammation.

This study presents several limitations. First, the study was conducted with a small number of participants, which could have selection bias. Second, we could not recruit male participants due to limited time available for them to take a 3-month exercise protocol. Third, we only used one exercise program protocol in this study. Multiple exercise protocols, such as exercise type, exercise period, and exercise intensity might have different effects on PM SUVmax. Fourth, the diet of participants was not fully controlled. Fifth, we could not acquire the tissue samples from PM, which could further support our conclusion. Finally, we were not able to control all confounding factors that might influence FDG distribution such as total image acquisition time and blood insulin levels.

Taken together, PM SUVmax evaluated by ^18^F-FDG PET/CT could reflect the obesity-driven inflammatory metabolic activity of skeletal muscle and chronic physical exercise reduced PM SUVmax and broke its relation with systemic inflammation. In spite of preliminary data, our findings could support the clinical benefits of chronic exercise on skeletal muscle glucose metabolism that might contribute to explain the significant reduction in new-onset diabetes in The Diabetes Prevention Program clinical trial [37]. Furthermore, PM SUVmax could be a promising surrogate marker for evaluation of obesity-induced skeletal muscle inflammation.

## Figures and Tables

**Table 1:** Baseline clinical characteristics of participants.

Baseline Characteristics	*n* = 23
Age (years)	46 ± 8.0
Height (cm)	156.8 ± 5.5
Smoking (current), *n* (%)	0 (0)
Alcohol drinking, *n* (%)	8 (34.8)
Menopause, *n* (%)	12 (52.2)
Hypertension (stage 1), *n* (%)	4 (17.4)
Diabetes, *n* (%)	0 (0)
Dyslipidemia, *n* (%)	7 (30.4)
Medication, *n* (%)	0 (0)

Age and height were shown as mean ± standard deviation.

**Table 2:** Comparison of clinical parameters between baseline and post-exercise.

Parameters	Baseline	Post-Exercise	*p*
Body weight (kg)	65.3 ± 7.5	62.4 ± 8	<0.001 *
BMI (kg/m^2^)	26.5 ± 2.2	25.3 ± 2.5	<0.001 *
Waist circumference (cm)	83.2 ± 5.5	81.3 ± 5.9	0.05 *
Hip circumference (cm)	98.6 ± 5.1	95.4 ± 4.6	<0.001 *
AST (IU/L)	12.3 ± 3.9	11.7 ± 5.1	0.47
ALT (IU/L)	21 ± 3.9	21 ± 4.2	0.69
Triglyceride (mg/dL)	105.7 ± 47.3	112.1 ± 46.6	0.26
Total cholesterol (mg/dL)	177.4 ± 30.6	178.3 ± 30	0.89
HDL-C (mg/dL)	49.9 ± 11.3	49.9 ± 9.2	0.71
LDL-C (mg/dL)	106.3 ± 29.5	101.3 ± 37.9	0.77
Glucose (mg/dL)	89 ± 8.3	88 ± 9.6	0.47
SBP (mmHg)	123.7 ± 16.4	116.4 ± 10.3	0.002 *
DBP (mmHg)	75 ± 11	71.6 ± 8	0.009 *
VAT area (cm^2^)	154.4 ± 32.7	142.3 ± 37.5	0.007 *
PM area (cm^2^)	18.5 ± 2.5	18.4 ± 2.3	0.402
hsCRP (mg/L)	1.98 ± 3.42	0.86 ± 1.34	0.006 *
Spleen SUVmax	1.73 ± 0.28	1.6 ± 3.21	0.004 *
BM SUVmax	1.55 ± 0.26	1.43 ± 0.22	0.018 *

All data were shown as mean ± standard deviation. BMI, body mass index; AST, aspartate transaminase; ALT, alanine aminotransferase; HDL-C, high-density lipoprotein cholesterol; LDL-C, low-density lipoprotein cholesterol; SBP, systolic blood pressure; DBP, diastolic blood pressure; VAT, visceral adipose tissue; PM, psoas muscle; hsCRP, high-sensitivity C-reactive protein; SUVmax, maximum standardized uptake value; BM, bone marrow. * Statistically significant difference.

**Table 3:** Correlations between PM SUVmax, PM area, and systemic inflammatory surrogate markers at baseline and post-exercise.

Status	Parameters	PM SUVmax	
		*r*	*p*
Baseline	hsCRP	0.611	0.002 *
	Spleen SUVmax	0.435	0.038 *
	BM SUVmax	0.315	0.145
	PM area (cm^2^)	−0.104	0.637
Post-exercise	hsCRP	−0.021	0.923
	Spleen SUVmax	0.075	0.735
	BM SUVmax	0.244	0.263
	PM area (cm^2^)	0.071	0.748

* Statistically significant difference. PM, psoas muscle; SUVmax, maximum standardized uptake value; hsCRP, high-sensitivity C-reactive protein; BM, bone marrow.

**Table 4:** Univariate- and multivariate analyses with PM SUVmax at baseline.

	Univariate		Multivariate	
Variable	Coefficients (95% CI)	*p*	*r*	*p*
Age	0.008 (−0.02–0.036)	0.549		
BMI (kg/m^2^)	0.15 (0.073–0.227)	0.001 *	0.177	0.468
Waist circumference (cm)	0.044 (0.008–0.08)	0.02 *	−0.032	0.897
Hip circumference (cm)	0.043 (0.002–0.083)	0.039 *	0.031	0.9
AST (IU/L)	−0.009 (−0.068–0.05)	0.752		
ALT (IU/L)	0.029 (−0.027–0.086)	0.292		
Triglyceride (mg/dL)	0.002 (−0.003–0.007)	0.445		
Total cholesterol (mg/dL)	0 (−0.007–0.008)	0.946		
HDL-C (mg/dL)	−0.01 (−0.03–0.009)	0.297		
LDL-C (mg/dL)	0.001 (−0.007–0.009)	0.797		
Glucose (mg/dL)	−0.003 (−0.031–0.024)	0.804		
SBP (mmHg)	0.006 (−0.008–0.019)	0.382		
DBP (mmHg)	0.012 (−0.008–0.032)	0.228		
VAT area (cm^2^)	0.004 (−0.003–0.011)	0.295		
PM area (cm2)	−0.047 (−0.135–0.042)	0.284		
Spleen SUVmax	0.798 (0.077–1.519)	0.032 *	0.347	0.145
BM SUVmax	0.278 (−0.579–1.135)	0.508		
hsCRP (mg/L)	0.111 (0.068–0.155)	<0.001 *	0.636	0.003 **

BMI, body mass index; AST, aspartate transaminase; ALT, alanine aminotransferase; HDL-C, high-density lipoprotein cholesterol; LDL-C, low-density lipoprotein cholesterol; SBP, systolic blood pressure; DBP, diastolic blood pressure; VAT, visceral adipose tissue; PM, psoas muscle; SUVmax, maximum standardized uptake value; BM, bone marrow; hsCRP, high-sensitivity C-reactive protein; CI, confidence interval. * Statistically significant difference. ** Statistically significant difference after Bonferroni correction (0.05/5). Multivariate analysis was performed after adjustment of age, AST, ALT, triglyceride, total cholesterol. HDL-C, LDL-C, glucose, SBP, DBP, VAT area, PM area, and BM SUVmax.

**Table 5:** Univariate analysis using PM SUVmax as dependent variable at post-exercise.

	Univariate	
Variable	Coefficients (95% CI)	*p*
Age	0 (−0.016–0.015)	0.956
BMI (kg/m^2^)	0.024 (−0.025–0.072)	0.325
Waist circumference (cm)	0.007 (−0.014–0.027)	0.508
Hip circumference (cm)	0.011 (−0.016–0.037)	0.406
AST (IU/L)	−0.001 (−0.026–0.024)	0.914
ALT (IU/L)	0 (−0.003–0.003)	0.753
Triglyceride (mg/dL)	−0.001 (−0.004–0.001)	0.321
Total cholesterol (mg/dL)	−0.00009 (−0.004–0.004)	0.965
HDL-C (mg/dL)	0.004 (−0.01–0.017)	0.576
LDL-C (mg/dL)	0.001 (−0.002–0.004)	0.587
Glucose (mg/dL)	−0.003 (−0.016–0.01)	0.662
SBP (mmHg)	0 (−0.013–0.012)	0.973
DBP (mmHg)	−0.003 (−0.019–0.013)	0.712
VAT area (cm^2^)	−0.001 (−0.004–0.002)	0.54
PM area (cm^2^)	0.006 (−0.047–0.06)	0.802
Spleen SUVmax	0.117 (−0.456–0.689)	0.676
BM SUVmax	0.287 (−0.268–0.841)	0.295
hsCRP (mg/L)	−0.031 (−0.141–0.079)	0.567

BMI, body mass index; AST, aspartate transaminase; ALT, alanine aminotransferase; HDL-C, high-density lipoprotein cholesterol; LDL-C, low-density lipoprotein cholesterol; SBP, systolic blood pressure; DBP, diastolic blood pressure; VAT, visceral adipose tissue; PM, psoas muscle; SUVmax, maximum standardized uptake value; BM, bone marrow; hsCRP, high-sensitivity C-reactive protein; CI, confidence interval.

## Data Availability

The data presented in this study are available on request from the corresponding author.
